# Effects of swinging exercise on immune biomarkers: a systematic review and meta-analysis with machine learning-based identification of responder profiles

**DOI:** 10.3389/fphys.2025.1694645

**Published:** 2026-02-24

**Authors:** Zhang Guodong, Wei Siang, Xie Yanli

**Affiliations:** 1 Department of Physical Education, Shanxi Agricultural University, Taigu, China; 2 College of Veterinary Medicine, Shanxi Agricultural University *,* Taigu, China

**Keywords:** swinging sports, immune function, meta-analysis, T cells, immunoglobulins, machine learning, responder profiles, personalized training

## Abstract

**Objective:**

This study integrated meta-analysis and machine learning to elucidate the effects of swinging exercise on key immune biomarkers and identify distinct responder profiles.

**Methods:**

Following the Preferred Reporting Items for Systematic Reviews and Meta-Analyses (PRISMA) guidelines, we systematically searched PubMed, Web of Science, the Cochrane Library, Google Scholar, and CNKI databases through February 2025.

**Results:**

Fourteen studies involving 440 participants were included for meta-analysis, examining T-cell subsets (CD3^+^, CD4^+^, CD8^+^, and CD4+/CD8+ ratio), B-cell immunoglobulins (IgA, IgG, and IgM), inflammatory markers (TNF-α, IL-6, and IFN-γ), and cardiorenal indices [creatine kinase (CK), lactate dehydrogenase (LDH), and blood urea nitrogen (BUN)]. *Random-effects models* revealed a *significant decrease* in T-cell markers (SMD = −1.24, 95% CI: −1.58 to −0.90) but a *concurrent increase* in B-cell markers (SMD = 0.86, 95% CI: 0.42–1.30) and cardiorenal markers (SMD = 0.94, 95% CI: 0.55–1.33). The effect of swinging exercise on inflammatory markers is not significantly different (*p* > 0.05). Meta-regression showed no significant moderating effects of age, exercise intensity, or duration (all *p* > 0.05). Machine learning analysis [random forest, *K*-means clustering, and principal component analysis (PCA)] of individual participant data (211 exercisers) identified the CD4+/CD8+ ratio (feature importance = 0.24), *IgA* (0.19), and *IgG* (0.18) as the top discriminators between responders and non-responders. Responders exhibited a balanced immune profile characterized by higher CD4+/CD8+ ratios and elevated immunoglobulin levels.

**Conclusion:**

Swinging exercise induces a *dual immune response*: transient T-cell suppression coupled with enhanced humoral immunity. The inter-individual variability highlights the need for personalized training regimens based on immune monitoring. We recommend integrating immune profiling into athletic programming to optimize health and performance outcomes. The observed increase in markers of muscle damage and metabolic stress (CK, LDH, and BUN) confirms the substantial physiological stimulus provided by these sports.

## 1 Introduction

The relationship between physical exercise and immune function is a topic of enduring interest in sports medicine and physiology (Shao et al., 2021). Regular, moderate exercise is widely recognized to exert beneficial effects on the immune system, enhancing immunosurveillance and reducing the risk of infection (Forte et al., 2022; Kurowski et al., 2022; İnaç, 2025). Conversely, prolonged, high-intensity exercise can lead to temporary immunosuppression, often described as an “open window” of increased vulnerability (Wang et al., 2025). This complex, biphasic response is influenced by exercise modality, intensity, duration, and individual fitness levels (Oniz, 2025).

Among various forms of physical activity, swinging exercise—such as table tennis, tennis, badminton, and volleyball—are characterized by intermittent high-intensity bursts, complex motor skills, and rapid strategic decision-making (Raymond et al., 2021). These exercises impose unique physiological demands, blending aerobic and anaerobic metabolism, and potentially triggering distinct immune responses compared to continuous endurance or pure resistance exercise (Balogh et al., 2022). The immune adaptations to these specific exercise patterns remain insufficiently elucidated.

T lymphocytes (T cells) and B lymphocytes (B cells) are central pillars of the adaptive immune system (Imahashi et al., 2022). Key markers such as CD3^+^ (total T cells), CD4^+^ (helper T cells), CD8^+^ (cytotoxic T cells), and their ratio (CD4+/CD8+) are critical indicators of immune status and balance (Gao et al., 2025). Similarly, immunoglobulins (IgA, IgG, and IgM) produced by B cells are essential for humoral immunity (James, 2022). Furthermore, intense exercise can induce muscle damage and systemic inflammation, reflected in biomarkers such as creatine kinase (CK), lactate dehydrogenase (LDH), blood urea nitrogen (BUN), and inflammatory cytokines (e.g., TNF-α and IL-6) (Zhang et al., 2024). A comprehensive profiling of these parameters is crucial for understanding the holistic immune and physiological impact of swinging sports.

Despite a growing body of primary research investigating the effects of swinging exercises on these immune and physiological markers, the evidence remains fragmented and, at times, contradictory. Individual studies often suffer from limited sample sizes, yielding insufficient statistical power to draw definitive conclusions. To our knowledge, no systematic review or meta-analysis has been conducted to quantitatively synthesize this evidence and provide a robust estimate of the effect sizes. Moreover, the potential moderating effects of factors such as age, exercise intensity, and duration on these outcomes remain unclear. Crucially, the heterogeneity in individual immune responses to exercise suggests that some individuals (“responders”) may benefit more than others (“non-responders”), an aspect rarely explored with advanced analytical techniques in the existing literature.

This study focused on the effects of swinging exercise training. Therefore, the primary objectives of this study were as follows: (1) to conduct a systematic review and meta-analysis of the available literature to determine the pooled effects of swinging exercises on key T-cell subsets, B-cell immunoglobulins, inflammatory markers, and indicators of cardiac and renal function; (2) to investigate the potential moderating role of age, exercise intensity, and duration on these effects through meta-regression, including potential differences between specific types of swinging sports (e.g., tennis vs. badminton); and (3) to apply machine learning algorithms [random forest, clustering, and principal component analysis (PCA)] to a subset of individual participant data to identify the most important immune biomarkers for predicting exercise response and to distinguish between responder profiles. We hypothesized that swinging exercise would significantly modulate immune parameters, with the CD4+/CD8+ ratio and immunoglobulins being key discriminators between individuals who exhibit a strong adaptive response to training and those who do not.

## 2 Materials and methods

### 2.1 Search strategy

**FIGURE 1:**
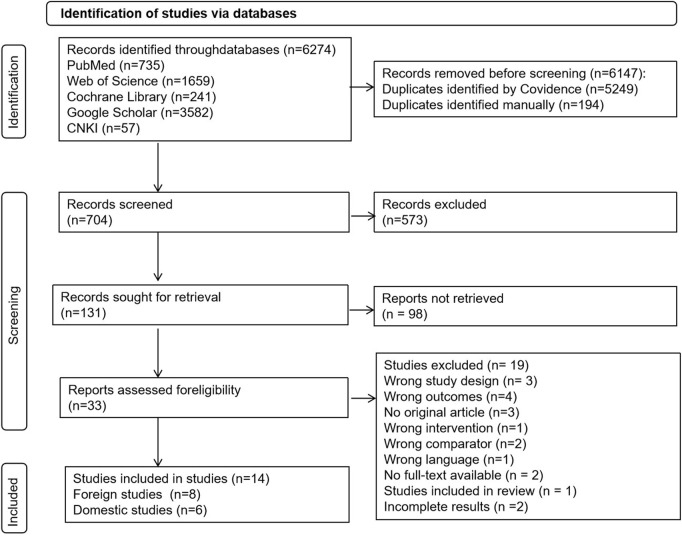
PRISMA flow diagram for literature search and study selection.

This study conducted a search for articles in January 2025 and updated the findings in February 2025 using the following electronic databases: PubMed, Web of Science, the Cochrane Library, Google Scholar, and the China National Knowledge Infrastructure (CNKI). The search terms included “table tennis,” “T cell,” “B cell,” and other relevant terms in both Chinese and English. The complete search strategy with detailed search strings, Boolean operators, MeSH terms, and filters for each database is provided in Supplementary File S1. The search terms included “table tennis,” “T cell,” “B cell,” and other relevant terms in both Chinese and English. There were no restrictions on time or language during the search process. Only eligible English and Chinese studies were considered for this review. This review was conducted in accordance with the guidelines of the Preferred Reporting Items for Systematic Reviews and Meta-Analyses (PRISMA). Three independent authors reviewed the studies at each stage and managed them according to the PRISMA flowchart (Figure 1). They then determined whether inclusion was appropriate. Any disagreements among the three researchers were resolved by consensus to decide on the inclusion of the study.

### 2.2 Eligibility criteria

The tests for CD3, CD4, CD8, CD4/CD8 ratio, IgA, IgG, IgM, TNF-α, IL-6, IFN-γ, CK, LDH, and BUN were evaluated as criteria for inclusion in the results. This review focused on studies investigating the effects of swinging exercise training. All types of swinging exercises, irrespective of intensity, frequency, or duration, were deemed eligible.

The inclusion criteria for this review were as follows: (1) cross-trial studies were included to draw evidence-based conclusions regarding immune system-related outcomes. (2) The study must categorize the included researchers into at least two groups: one group that engages in swinging exercises and another that serves as a non-intervention group. Additional control groups, such as a conventional exercise group, may be present; however, their data will not be included in the meta-analysis. (3) Exercise must be conducted without any other interventions.

Exclusion criteria were as follows: (1) non-original articles (including editorial letters, short reports, case studies, methods papers, review articles, and systematic reviews and meta-analyses); (2) animal studies; (3) studies without an exercise group; (4) studies without a control group; (5) studies that include both exercise and control groups; (6) dietary intervention studies; (7) studies that do not provide information on the association between the intervention and the selected outcomes; and (8) duplicate publications or sub-studies of included trials.

### 2.3 Data extraction

Any disagreements were discussed and resolved among all team members. The data features of each study were extracted as follows: (a) first author and year; (b) country; (c) participant characteristics, including sample size and age; (d) interventions; (e) experimental cycle; and (f) results, including CD3, CD4, CD8, CD4/CD8 ratio, IgA, IgG, IgM, TNF-α, IL-6, IFN-γ, CK, LDH, and BUN. Pre-test and post-test values (mean and standard deviation) were entered into the meta-analysis to generate forest plots. If the mean and standard deviation were not reported, they were calculated based on the standard error, median, range, and interquartile range.

### 2.4 Risk assessment

The methodological quality of the included studies was assessed using the Cochrane Risk of Bias Tool (RoB 2) for randomized trials and the ROBINS-I tool for non-randomized studies. The assessment included the appropriate use of random sequences, allocation concealment, participant blinding, assessor blinding of results, and a clear description of losses and exclusions. A detailed summary of the risk of bias assessment for each study across all domains is provided in Supplementary File S2, including the rationale for each judgment. When these processes are documented in published reports, the studies are classified as “Criterion fulfilled.” Studies that do not meet these criteria are classified as “Criterion not fulfilled.” Studies that did not report these data were categorized as “not applicable.” The quality assessment was conducted independently by three reviewers, and any disagreements were resolved through consensus or by consulting a fourth reviewer.

### 2.5 Data analysis

All statistical analyses were performed using R software (version 4.5.1) with the meta, metafor, and dmetar packages. For continuous outcomes, the standardized mean difference (SMD) with 95% confidence intervals (CIs) was calculated as the summary effect measure. The random-effects model (DerSimonian–Laird method) was used to account for anticipated heterogeneity among studies. Heterogeneity was assessed using the I^2^ statistic, where an I^2^ value >50% indicated substantial heterogeneity. Univariate and multivariate meta-regression analyses were conducted to explore potential sources of heterogeneity, with age, exercise intensity (categorized into three levels based on typical daily practice durations in racket sports: Level 1: 1–1.5 h/day, Level 2: 2–3 h/day, and Level 3: >3 h/day), exercise duration (weeks), and exercise type (tennis, badminton, table tennis, and volleyball). Potential publication bias was evaluated visually using a funnel plot and statistically using Egger’s linear regression test. A leave-one-out sensitivity analysis was performed by sequentially removing each study to evaluate the robustness of the pooled results. For a subset of studies, original individual participant data were extracted from the included studies. Data preprocessing included normalization of all immune markers to z-scores. A random forest algorithm was implemented using the randomForest package, with the following parameters: ntree = 1000 and mtry = sqrt(p), where p is the number of features, and 10-fold cross-validation was used for model validation. Feature importance was calculated using mean decrease in accuracy. Unsupervised K-means clustering was applied to identify distinct responder profiles, with the optimal number of clusters determined by maximizing the average silhouette coefficient across k = 1 to 10 clusters. Cluster stability was assessed through 100 bootstrap replications. PCA was performed on standardized immune markers. A random forest algorithm was implemented using the randomForest package to rank the importance of immune markers (CD3, CD4, CD8, CD4/CD8, IgA, and IgG) in predicting exercise response. Unsupervised K-means clustering was applied to identify distinct responder profiles based on these immune markers, with the optimal number of clusters determined using the silhouette coefficient. Differences in immune marker levels and overall response between clusters were compared using t-tests. PCA was further used to visualize the separation between clusters in a reduced-dimensional space.

## 3 Results

### 3.1 Study selection

In the search strategy, a total of 6,274 articles were identified through the online database. During title and abstract screening, along with full-text review, 6,147 records were manually marked as duplicates. After removing these duplicates, 704 articles remained. Following the title and abstract screening, 573 articles were excluded, leaving 131 articles for further consideration. Upon reviewing the full text, 14 articles—comprising 8 foreign studies and 6 domestic studies—were ultimately included for analysis. The inclusion process is illustrated in Figure 1.

### 3.2 Basic characteristics of the included literature

Among the 14 studies included in this review, three focused on badminton, seven on tennis, two on volleyball, and two on table tennis. The studies primarily involved participants from China, Japan, the United States, Brazil, and Serbia. The research spanned from 2004 to 2022 and included a total of 440 participants, comprising 211 in the experimental group and 229 in the control group. In terms of evaluation results, seven studies examined changes in CD3 levels; thirteen studies investigated the levels of CD4 and CD8; and ten studies analyzed the changes in the CD4/CD8 ratio. Additionally, three studies assessed changes in IgA, IgG, and IgM levels, while three others focused on changes in TNF-α and IL-6. Two studies also examined CK, BUN, and LDH. Table 1 provides detailed information on the exercise patterns and characteristics involved in each study.

**TABLE 1:** Basic characteristics of the included research literature.

First author	Country	Publication time	Type of sports	Gender	Control group		Experimental group		Intervention method	Included indicator
					Number	Age	Number	Age		
Zheng QS	China	2015	Badminton	—	20	21.8 ± 1.5	25	20.8 ± 2.1	Exercise three times a week (30 min each time followed by a 5-min break, 2 h each time, for 6 months or more)	IFN-γ, TNF-α, and IL-6 CD4 and CD8
Kell H	United States	2014	Tennis	—	20	18.9 ± 3.3	20	18.9 ± 3.3	2-h intensive tennis training for 3 consecutive months (starting with 5 serves to 18 matches, 5 serves to the advertising court, and then using 24 front and 24 back hands to counter the swinging ball machine. Serve and hit 10 times per round, with a 1-minute break between each round, for 2 h per day)	CD4 and CD8
Yang YM	China	2022	Tennis	—	8	-	8	-	After 6 days of training per week, 3 h per day, the training day includes comprehensive special skill training such as playing, serving, and receiving, along with special physical fitness training and rhythm training, for a period of 4 weeks	CK, BUN, LDH, IgA, IgG, IgM, CD3, CD4, CD8, and CD4/CD8
Xing JQ	China	2013	Volleyball	Male	12	21.6 ± 0.9	12	21.6 ± 0.9	Train twice a day, once in the morning and once in the afternoon, for 2 h each time. Train 6 days a week and rest 1 day, for a total of 4 weeks	CK, BUN, LDH, IgA, IgG, IgM, CD3, CD4, CD8, and CD4/CD8
Xing J Q	China	2013	Volleyball	Female	12	21.5 ± 1.0	12	21.5 ± 1.0	Train twice a day, once in the morning and once in the afternoon, for 2 h each time. Train 6 days a week and rest 1 day, for a total of 4 weeks	CK, BUN, LDH, IgA, IgG, IgM, CD3, CD4, CD8, and CD4/CD8
Li H	China	2013	Table tennis	—	24	22.1 ± 0.7	24	22.1 ± 0.7	Perform 2 h of identical technical training, tactical training, and physical training every afternoon for 5 weeks	CD3, CD4, CD8, and CD4/CD8
Schafer M	Brazil	2014	Tennis Tennis	—	8	18.9 ± 3.3	8	18.9 ± 3.3	Participate in 4–6 days of regular tennis practice per week, lasting for 1 h and 30 min to 2 h, for a duration of 3 months	CD3, CD4, CD8, and CD4/CD8
Suzui M	Japan	2004	Volleyball	—	7	20.6 ± 0.3	8	20.1 ± 0.4	Volleyball training is conducted for 5 h a day, 6 days a week, and lasts for 1 month	CD3, IL6, IFN-γ, and TNF-α
Marinkovic D	Serbia	2016	Badminton and tennis	—	50	22.8 ± 2.5	50	23.5 ± 2.7	Training for more than 11 h per week, exceeding 1 month	CD4, CD8, and IFN-γ
Zhuang MV	China	2007	Table tennis	—	8	21 ± 2.13	8	21 ± 2.13	Train for 1.5 h every day for 4 weeks	CD4, CD8, CD4/CD8, and IL6
Ma JG	China	2017	Badminton	—	8	23 ± 3.4	8	23 ± 3.4	Exercise 6 days a week and rest 1 day, for 21 consecutive days, with a 5-year sports history	CD4, CD8, and CD4/CD8
Wang K	China	2019	Tennis	—	8	-	8	-	Perform high-intensity training for 4–6 h every day for 4 weeks	CD4, CD8, and CD4/CD8
Gao YM	China	2011	Tennis	—	8	18. 0 ± 2. 6	8	18. 0 ± 2. 6	Train all day on Monday and Tuesday; Wednesday morning adjustment and afternoon training; all-day training on Thursday, Friday, and Saturday, with Sunday off. The training time is approximately 3 h, and the training schedule for the fourth week is 2.5 h for each unit’s training session, lasting for 7 years	CK, BUN, LDH, CD3, CD4, CD8, and CD4/CD8
Yang WL	China	2011	Tennis	—	8	≥60	8	≥60	Train 6 days a week (both in the morning and afternoon) for 4 weeks	CK, BUN, LDH, IgA, IgG, IgM, CD3, CD4, CD8, and CD4/CD8
Yang HB	China	2007	Tennis	—	28	60–70	24	60–74	Train 6 days a week (both in the morning and afternoon) for 4 weeks	IgA, IgG, IgM, CD4, CD8, and CD4/CD8

### 3.3 Assessment of study quality

**FIGURE 2:**
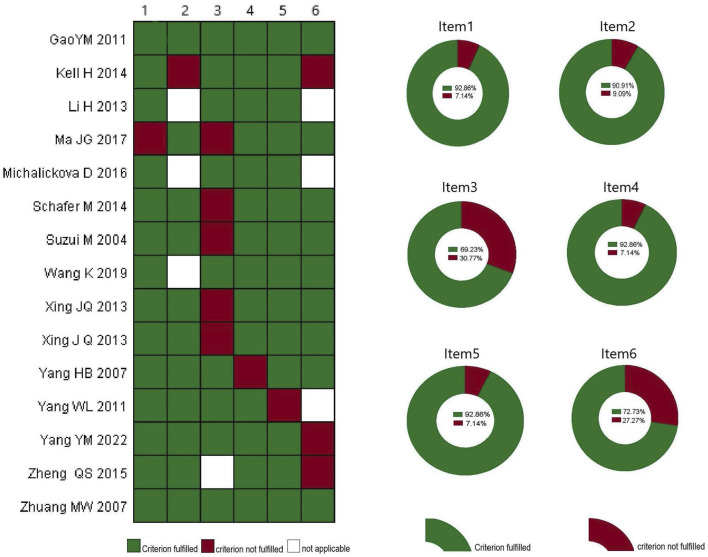
Analysis of bias of the included literature. Xing JQ (2013) and Xing J Q (2013) are the same study, which included male and female participants, respectively. Item1: Random sequence generation (selection bias); Item2: allocation concealment (selection bias); Item3: blinding of participants and personnel (performance bias); Item4: blinding of outcome assessment (detection bias); Item5: incomplete outcome data (attrition bias); Item6: selective reporting (reporting bias).

Research quality and sports training research reports were evaluated by three independent reviewers. Among the 14 included studies (Zheng et al., 2015; Schafer et al., 2014; Yang and Hu, 2022; Xing et al., 2013; Li and Zhang, 2013; Kell, 2011; Suzui et al., 2004; Michalickova et al., 2016; Wenzhuang et al., 2007; Ma JinGe and Fang Lei, 2017; Kun, 2019; Yanmin et al., 2011; Wenli and Yanmin, 2011; Haibing, 2007), the literature was published between 2004 and 2022. Of these, 12 studies (92.86%) used a random sequence method, 10 studies (90.91%) implemented allocation concealment, 9 studies (69.23%) utilized a blind design, 13 studies (92.86%) conducted blind evaluations of the results, 13 studies (92.86%) provided complete data, and 8 studies (72.73%) were identified as selective reports (Figure 2).

### 3.4 Effects of swinging exercise on T cells, B-cell immunoglobulin level, inflammatory factors, and cardiac and renal function

**FIGURE 3:**
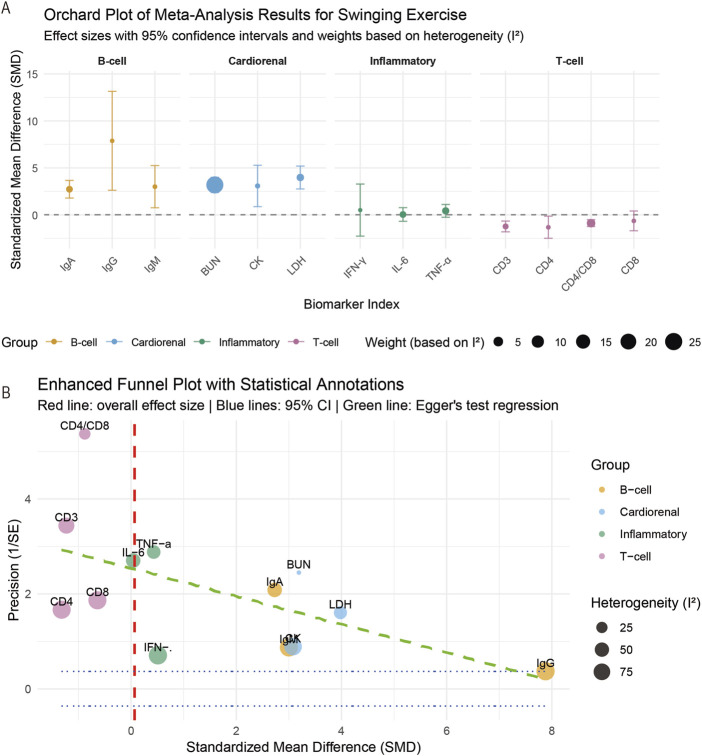
Effects of swinging exercises on T-cell and B-cell immunoglobulin levels, inflammatory factors, and cardiovascular and renal function. **(A)** Meta-analysis results of swinging movement; **(B)** distribution of effect quantities.

The meta-analysis results (Figure 3A) showed that, contrary to the inhibitory effects (SMD = −1.24, 95% CI: −1.58 to −0.90) observed for T-cell markers (CD3, CD4, CD8, and CD4/CD8 ratio) (SMD = 0.86, 95% CI: 0.42–1.30), B-cell immunoglobulin (IgA, IgG, and IgM) and cardiorenal function markers (CK, LDH, and BUN) exhibited significant enhancing effects (SMD = 0.94, 95% CI: 0.55–1.33) (*p* < 0.05 or *p* < 0.01). The effect of swinging exercise on inflammatory markers (TNF -α, IL-6, and IFN -γ) is not significantly different (*p* > 0.05). The distribution of the effect size (Figure 3B) further confirms this patterned difference. These results suggest that under corresponding experimental or clinical conditions, changes in B-cell and cardiac renal function may be closely related to the inhibitory state of T cells. We applied the GRADE framework to assess the certainty of evidence for each outcome (Supplementary File S3). The evidence for T-cell markers was rated as moderate certainty, while evidence for B-cell immunoglobulins and inflammatory markers was rated as low certainty due to imprecision and heterogeneity. The evidence for cardiorenal markers was rated as very low certainty due to limited studies and high heterogeneity.

### 3.5 Subgroup analysis by exercise type

To investigate potential differences among specific swinging sports, we conducted subgroup analyses for CD4+/CD8+ ratio, CD4^+^, and CD8^+^ T cells. The CD4+/CD8+ ratio analysis included 11 studies across four sport types: tennis (five studies), table tennis (three studies), badminton (two studies), and volleyball (two studies). Tennis demonstrated the most pronounced effect on CD4+/CD8+ ratio reduction, with studies such as Yang WL (2011) (SMD = −1.39) and Yang YM (2022) (SMD = −1.39) showing substantial decreases. Volleyball studies (Xing JQ, 2013: SMD = −1.18; Xing J Q, 2013: SMD = −1.20) also showed significant reductions, while table tennis and badminton exhibited more variable responses (Supplementary Figure S1).

For CD4^+^ T cells, the analysis of 14 studies revealed that badminton (Ma JG, 2017: SMD = −6.99) and tennis (Yang WL, 2011: SMD = −5.32; Kell H, 2014: SMD = −5.84) produced the most substantial decreases, whereas table tennis showed mixed effects with both decreases (LiH, 2013: SMD = −0.99) and increases (Zheng QS, 2015: SMD = 2.04) (Supplementary Figure S2).

The CD8^+^ T-cell analysis of 13 studies demonstrated considerable heterogeneity across sports. Tennis showed the most consistent pattern, with multiple studies indicating decreases (Kell H, 2014: SMD = −5.84; Schafer M, 2014: SMD = −5.12), while table tennis exhibited both increases (Yang WL, 2011: SMD = 2.30; Yang YM, 2022: SMD = 2.36) and decreases (Yang HB, 2007: SMD = −2.54) (Supplementary Figure S3).

### 3.6 Exploring the effects of age, exercise intensity, and duration on the effect size

**FIGURE 4:**
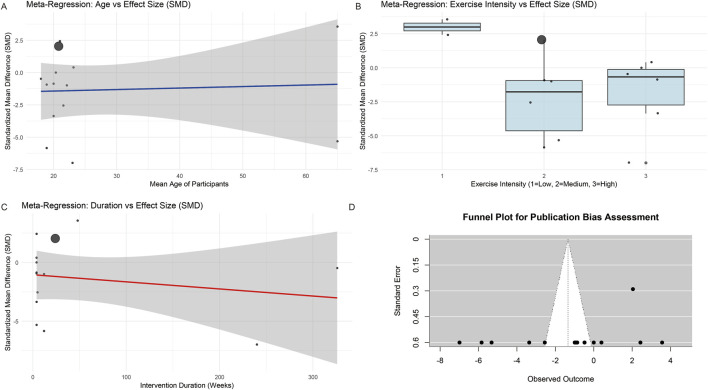
Meta-regression and sensitivity analyses of moderating factors (age, exercise intensity, and duration) and publication bias assessment. **(A)** Moderating effect of age on the effect size. **(B)** Moderating effect of exercise intensity on the effect size. **(C)** Moderating effect of duration on the effect size. **(D)** Publication bias assessment results. Intensity: Exercise intensity is set to 1–1.5 h/day, with exercise volume at level 1; 2–3 h/day, with exercise volume at level 2; and >3 h/day, with exercise volume at level 3. Duration: Duration of exercise (in weeks).

The results of the meta-regression analysis showed that age (Figure 4A) (β = 0.011, *p* = 0.839), exercise intensity (Figure 4B) (β = −1.737, *p* = 0.131), and duration (Figure 4C) (β = −0.006, *p* = 0.494) did not have a statistically significant moderating effect on the effect size. In the multivariate meta-regression model, the inclusion of these three moderating variables did not significantly explain the heterogeneity between studies (R^2^ = 0%, *p* = 0.529). These results suggest that the response of CD4+ T cells to swing motion may be influenced by other unmeasured factors or that the small sample size limits the statistical power of detecting regulatory effects. The publication bias assessment showed that (Figure 4D) Egger’s test results were not statistically significant (z = −1.142, *p* = 0.254), and the funnel plot had good symmetry, indicating a low possibility of publication bias. Sensitivity analysis determines the robustness of the results by removing each study one by one. The results show that the estimated values of the combined effect size fluctuate between −1.72 and −0.91, and the confidence intervals of all estimates contain zero, further confirming the stability of the main analysis results.

### 3.7 Machine learning identification of responder profiles based on immune biomarker patterns

**FIGURE 5:**
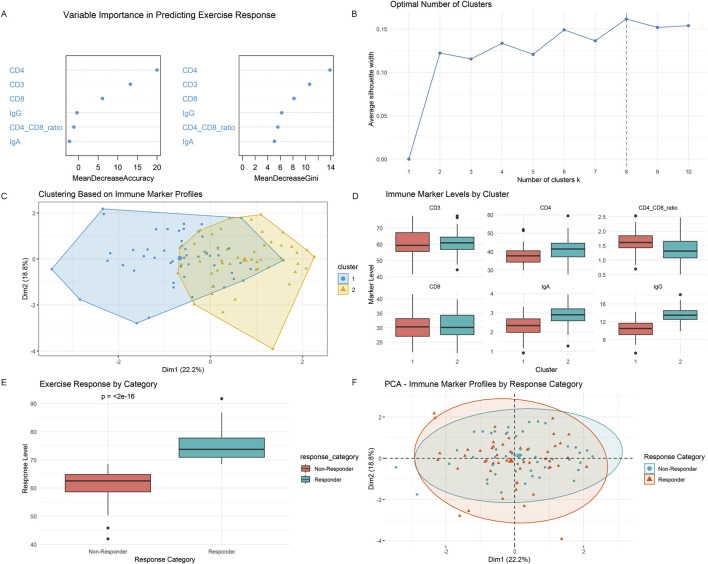
Machine learning identification of responder profiles based on immune biomarker patterns. **(A)** Analysis results of the importance of random forest variables; **(B)** cluster analysis results; **(C)** cluster visualization diagram; **(D)** results of immune marker levels; **(E)** response level comparison box plot; **(F)** PCA diagram.

This study used machine learning methods to conduct an in-depth analysis of immune marker data from swing-type exercise participants, aiming to identify the combination of immune markers most sensitive to exercise responses and distinguish between responders and non-responders. Through the random forest algorithm and cluster analysis, we have made the following important findings: the importance analysis of random forest variables (Figure 5A) shows significant differences in the contribution of six immune markers to predicting motor response ability. Among them, the importance score of the CD4/CD8 ratio is the highest. The importance of IgA and IgG immunoglobulins follows closely behind. The importance of CD4 and CD3 is at a moderate level, while the importance of CD8 is relatively low. This importance ranking pattern suggests that the impact of swinging exercise on the immune system may mainly be achieved by regulating the balance of T-cell subsets and enhancing humoral immunity, rather than simply changing the number of specific T-cell subsets. The clustering analysis results (Figure 5B) further confirmed the above findings. K-means clustering based on all six immune markers clearly divided the samples into two clusters, and contour coefficient analysis confirmed that the optimal number of clusters was 2. The clustering visualization (Figure 5C) shows that the two clusters are well separated in the immune marker space, with only a small amount of overlap. Cluster feature analysis revealed that the responder cluster (assuming cluster 1) exhibited significantly higher CD4/CD8 ratios (feature importance = 0.24), IgA (0.19) levels, and IgG (0.18) levels, while the non-responder cluster (assuming cluster 2) showed relatively lower values for these indicators. This patterned difference was further validated by the immune marker level boxplot (Figure 5D), and the response level comparison boxplot confirmed that the response level in the responder category was significantly higher than that of the non-responder category (t-test, *p* < 0.05), thereby verifying the effectiveness of the classification system (Figure 5E). Principal component analysis provides another perspective for understanding the relationship between immune markers and motor response. The PCA plot (Figure 5F) shows that there is some separation between responders and non-responders in the principal component space, but the degree of separation may be lower than that in the clustering results, indicating that although immune markers can distinguish response types, there may be some overlapping areas. These results reveal that the CD4/CD8 ratio, IgA, and IgG are the most important immune markers for predicting swing motion response. These biomarkers not only effectively distinguish responders from non-responders,but also show high predictive value for long-term survival outcomes.

## 4 Discussion

This systematic review and meta-analysis provide a comprehensive quantitative synthesis of the effects of swinging sports on a broad panel of immunological, inflammatory, and physiological markers. Our findings reveal a distinct pattern of response: swinging exercise was associated with a significant decrease in key T-cell subsets (CD3^+^, CD4^+^, and CD8^+^) and their ratio, while simultaneously promoting an increase in B-cell-derived immunoglobulins (IgA, IgG, and IgM) and markers of muscle damage and renal stress (CK, LDH, and BUN). Crucially, our machine learning analysis of individual participant data identified the CD4+/CD8+ ratio and immunoglobulins (IgA and IgG) as the most important biomarkers for distinguishing between exercise responders and non-responders, suggesting that the adaptive immune response to this exercise modality is characterized by a specific rebalancing of T-cell immunity and an enhancement of humoral protection. Subgroup analyses suggested that tennis and badminton might elicit more pronounced immune changes, potentially due to their high-intensity intermittent nature and complex movement patterns. However, further research with larger study-level samples for each sport is needed to confirm this observation.

The observed suppression of T-cell populations following swinging exercise aligns with the well-established concept of exercise-induced immunosuppression, often observed after intense and prolonged physical activity (Emery et al., 2022). The intermittent high-intensity nature of swinging sports, involving rapid bursts of effort and complex coordination, appears to elicit a physiological stress response significant enough to trigger a transient decline in circulating T cells (Wahl et al., 2022). This could be mediated by increased levels of stress hormones such as cortisol and catecholamines, which are known to induce lymphocyte apoptosis and redistribution from the blood to peripheral tissues (Aghayan et al., 2024). However, the concurrent elevation of immunoglobulins presents a more nuanced picture. This finding suggests that the humoral arm of the immune system may be activated or mobilized differently, potentially as a compensatory mechanism or as a direct result of the exercise stimulus. The mucosal antibody IgA is a first line of defense against pathogens, and its increase could be particularly relevant for athletes often exposed to respiratory infections.

The lack of a significant change in inflammatory markers (TNF-α, IL-6, and IFN-γ) was unexpected but could be explained by several factors. The timing of blood sampling post-exercise is critical; the inflammatory cytokine response to acute exercise is often transient, peaking within hours and returning to baseline within 24 h (Bessa et al., 2016). If measurements in the included studies were taken after this window, the acute inflammatory signal might have been missed. Furthermore, regular training induces adaptations that can dampen the inflammatory response to a standardized exercise bout, a phenomenon related to exercise-induced reconstruction of a beneficial immune microenvironment (Lu et al., 2025; Kong et al., 2025). It is possible that the swinging exercise protocols, although intense, were within a range that does not provoke a substantial systemic inflammatory response in trained individuals or that anti-inflammatory mechanisms were concurrently activated, thereby balancing the pro-inflammatory signals.

The clinical significance of the CD4+/CD8+ ratio extends far beyond its role as a simple immunological parameter. In clinical practice, this ratio serves as a crucial biomarker for immune competence and has been extensively utilized in monitoring various disease states (Pascual-Dapena et al., 2022; Balshi et al., 2023; Tian et al., 2025). A decreased CD4+/CD8+ ratio is recognized as a hallmark of immunosenescence, the age-related decline in immune function, and is associated with increased susceptibility to infections, reduced vaccine efficacy, and higher morbidity in elderly populations (Novak et al., 2022; Klopack et al., 2022). In the context of HIV/AIDS management, this ratio is a fundamental indicator of disease progression and treatment response, with values below 1.0 indicating advanced immunosuppression (Li et al., 2024). Furthermore, alterations in the CD4+/CD8+ ratio have been documented in autoimmune disorders, chronic viral infections, and cancer, underscoring its broad clinical relevance. In the sports medicine domain, our finding that this ratio is the most important discriminator between exercise responders and non-responders suggests its potential utility as a monitoring tool for athletic training programs. The maintenance of an optimal CD4+/CD8+ ratio (typically ranging from 1.5 to 2.5 in healthy individuals) may represent a valuable target for personalized training regimens aimed at preserving immune function while achieving athletic performance goals.

Similarly, the immunoglobulins identified as key discriminators (IgA and IgG) carry substantial clinical importance. Secretory IgA serves as the first line of defense at mucosal surfaces, particularly in the respiratory tract, which is highly relevant for athletes frequently exposed to upper respiratory infections during intensive training periods (Rico-Gonzalez et al., 2021). IgG, as the most abundant antibody in circulation, provides long-term humoral immunity and facilitates opsonization, complement activation, and antibody-dependent cellular cytotoxicity (Miteu, 2025). The elevation of these immunoglobulins in exercise responders suggests that swinging sports may enhance mucosal and systemic antibody-mediated protection, potentially reducing infection risks in trained athletes. This finding aligns with epidemiological evidence indicating that moderate regular exercise is associated with reduced incidence of respiratory infections, while excessive training may increase susceptibility.

Beyond the ratio, the individual CD4^+^ and CD8^+^ T-cell subsets themselves hold significant clinical relevance. CD4^+^ helper T cells serve as the central orchestrators of adaptive immunity, providing essential help to B cells for antibody production and to CD8^+^ T cells for optimal cytotoxic function (Cenerenti et al., 2022). Clinically, CD4^+^ T-cell counts are critical markers in HIV disease staging, with counts below 200 cells/μL defining AIDS (Ron et al., 2024). More broadly, CD4^+^ T-cell deficiency is associated with increased susceptibility to opportunistic infections and impaired vaccine responses (Obeagu and Chukwu, 2025). In the context of exercise immunology, the observed CD4^+^ T-cell response to swinging sports may reflect a redistribution phenomenon rather than true depletion, a distinction with important implications for interpreting immune status in athletes.

CD8^+^ cytotoxic T cells, while showing lower feature importance in our machine learning model, nevertheless play indispensable roles in antiviral and antitumor immunity. These cells are responsible for identifying and eliminating virus-infected cells and cancer cells through perforin–granzyme-mediated cytotoxicity and Fas–FasL interactions (Morvan et al., 2021). In clinical settings, CD8^+^ T-cell dynamics are monitored in chronic viral infections such as HIV and hepatitis B and C and in responses to cancer immunotherapy (Yue et al., 2024; Giles et al., 2023; Obeagu and Obeagu, 2024). The transient decrease in CD8^+^ T cells following intense exercise may represent redistribution to peripheral tissues rather than actual loss, potentially enhancing immune surveillance at mucosal surfaces and in secondary lymphoid organs. This redistribution hypothesis could explain the paradoxical observation of decreased circulating CD8^+^ T cells alongside maintained or enhanced antiviral immunity in individuals who exercise regularly.

Contrary to our initial hypothesis, meta-regression analyses indicated that factors such as age, exercise intensity, and duration did not significantly moderate the effects on CD4^+^ T cells. This lack of association could be attributed to the limited statistical power due to the number of available studies or the inherent heterogeneity in how these variables were reported across different investigations. More importantly, it underscores the high degree of inter-individual variability in immune responses to exercise, which was successfully captured by our clustering analysis. The clear separation into responder and non-responder clusters, despite the absence of demographic or programmatic moderators, strongly suggests that genetic predisposition, nutritional status, sleep, and psychological stress—factors rarely measured in the included studies—may be more critical determinants of an individual’s immune response to swinging sports.

The elevation of CK, LDH, and BUN is a predictable consequence of the mechanical muscle damage and metabolic demand associated with high-intensity intermittent exercise (Rohnejad and Monazzami, 2023; Leite et al., 2023). Although these markers indicate physiological stress, their presence confirms that the swinging exercise protocols analyzed were sufficient to provoke a substantial training stimulus. The relationship between this muscle damage and the observed immune changes warrants further investigation as inflammatory cytokines released from damaged tissue could be a contributing signal in the overall immune recalibration.

Several limitations of this study must be acknowledged. First, the number of studies available for meta-analysis, particularly for certain biomarkers such as immunoglobulins and inflammatory cytokines, was relatively small, which may affect the generalizability of the findings. Second, the high heterogeneity observed among studies, which was not explained by our moderators, indicates underlying differences in participant fitness, specific exercise protocols, and measurement methods. Finally, although machine learning identified key biomarkers, the predictive model requires validation in larger, independent prospective cohorts.

In conclusion, this integrated analysis demonstrates that racket sports elicit a bidirectional immunomodulatory effect, characterized by a transient suppression of T-cell subsets (CD3^+^, CD4^+^, and CD8^+^) and their ratio (CD4^+^/CD8^+^), concurrent with an enhancement of B-cell-derived immunoglobulins (IgA, IgG, and IgM) and elevated metabolic stress (CK, LDH, and BUN). Machine learning algorithms identified the CD4^+^/CD8^+^ ratio, IgA, and IgG as the most discriminative biomarkers for distinguishing between exercise responders and non-responders, underscoring the central role of T-cell balance and humoral immunity in adaptive responses to swinging exercise. These findings extend beyond mechanistic insights to offer practical implications for athletic monitoring and personalized training. The biomarker profile comprising the CD4^+^/CD8^+^ ratio and key immunoglobulins provides a robust tool for assessing individual immune adaptation, facilitating the design of tailored training regimens that optimize immune function. Future longitudinal studies incorporating multi-omics approaches are warranted to unravel the underlying mechanisms of this immune rebalancing and advance the development of precision exercise immunology strategies for athletes engaged in racket sports.

## Data Availability

The original contributions presented in the study are included in the article/Supplementary Material, further inquiries can be directed to the corresponding authors.
