# Supplementary material for: Effects of swinging exercise on immune biomarkers: a systematic review and meta-analysis with machine learning-based identification of responder profiles
Source: Front Physiol. 2026 Feb 24;16:1694645. doi: 10.3389/fphys.2025.1694645 (PMC12973063; doi:10.3389/fphys.2025.1694645)
Supplement: Supplementary file 1 [file Supplementaryfile2.doc]

**Supplementary File S2: Detailed Risk of Bias Assessment**

Assessment Tools:

Cochrane Risk of Bias Tool (RoB 2) for randomized trials

ROBINS-I Tool for non-randomized studies

Judgment Categories: Low risk of bias, Some concerns, High risk of bias, No information.

### **Randomized Studies (RoB 2 Assessment)：**

| **Study ID** | **Randomization Process** | **Deviations from Intended Interventions** | **Missing Outcome Data** | **Outcome Measurement** | **Selective Reporting** | **Overall Bias** |
| --- | --- | --- | --- | --- | --- | --- |
| Zheng QS 2015 | Low risk of bias | Low risk of bias | Low risk of bias | **Some concerns** | Low risk of bias | **Some concerns** |
| Kell H 2014 | Low risk of bias | Low risk of bias | Low risk of bias | Low risk of bias | Low risk of bias | **Low risk** |
| Yang YM 2022 | **Some concerns** | Low risk of bias | Low risk of bias | **Some concerns** | Low risk of bias | **Some concerns** |
| Li H 2013 | Low risk of bias | Low risk of bias | Low risk of bias | Low risk of bias | Low risk of bias | **Low risk** |
| Schafer M 2014 | Low risk of bias | Low risk of bias | Low risk of bias | Low risk of bias | Low risk of bias | **Low risk** |

### **Non-Randomized Studies (ROBINS-I Assessment)：**

| Study ID | Confounding | Participant Selection | Intervention Classification | Deviations from Interventions | Missing Data | Outcome Measurement | Selective Reporting | **Overall Bias** |
| --- | --- | --- | --- | --- | --- | --- | --- | --- |
| Xing JQ 2013 | **Some concerns** | Low risk of bias | Low risk of bias | Low risk of bias | Low risk of bias | High risk of bias | Low risk of bias | **High risk** |
| Xing J Q 2013 | **Some concerns** | Low risk of bias | Low risk of bias | Low risk of bias | Low risk of bias | High risk of bias | Low risk of bias | **High risk** |
| Suzui M 2004 | High risk of bias | Low risk of bias | Low risk of bias | Low risk of bias | Low risk of bias | Low risk of bias | Low risk of bias | **Moderate risk** |
| Marinkovic D 2016 | High risk of bias | Low risk of bias | Low risk of bias | Low risk of bias | Low risk of bias | High risk of bias | Low risk of bias | **Moderate risk** |
| Ma JG 2017 | **Some concerns** | Low risk of bias | Low risk of bias | Low risk of bias | Low risk of bias | High risk of bias | Low risk of bias | **High risk** |
| Wang K 2019 | **Some concerns** | Low risk of bias | Low risk of bias | Low risk of bias | Low risk of bias | High risk of bias | Low risk of bias | **High risk** |
| Gao YM 2011 | High risk of bias | Low risk of bias | Low risk of bias | Low risk of bias | Low risk of bias | High risk of bias | Low risk of bias | **Moderate risk** |
| Yang WL 2011 | **Some concerns** | Low risk of bias | Low risk of bias | Low risk of bias | Low risk of bias | High risk of bias | Low risk of bias | **High risk** |
| Yang HB 2007 | **Some concerns** | Low risk of bias | Low risk of bias | Low risk of bias | Low risk of bias | High risk of bias | Low risk of bias | **High risk** |

Rationale for Judgments

Domain 1: Randomization Process (RoB 2)

Low risk:

Zheng QS 2015: "Computer-generated random sequence"

Kell H 2014: "Random number table used"

Li H 2013: "Block randomization with sealed envelopes"

Some concerns:

Yang YM 2022: "Randomization mentioned but method not specified"

Domain 2: Confounding (ROBINS-I)

High risk:

Xing JQ 2013: "No adjustment for age, fitness level, or baseline immune status"

Ma JG 2019: "Significant baseline differences in training history not accounted for"

Moderate risk:

Suzui M 2004: "Partial adjustment for age but not for other potential confounders"

Domain 3: Participant Selection

Low risk:

All studies: Clear inclusion/exclusion criteria reported

Domain 4: Intervention Classification

Low risk:

All studies: Swinging exercise interventions clearly described

Domain 5: Deviations from Intended Interventions

Low risk:

Majority of studies: Protocol adherence monitored and reported

Domain 6: Missing Data

Low risk:

Most studies: Complete outcome data or appropriate handling of missing data

Domain 7: Outcome Measurement

Some concerns:

Multiple studies: Laboratory methods for immune markers described but blinding of assessors not specified

High risk:

Yang HB 2007: "Subjective assessment of some immune parameters without validation"

Domain 8: Selective Reporting

Low risk:

Most studies: All pre-specified outcomes reported

Some concerns:

Wang K 2019: "Multiple inflammatory markers measured but only selected results reported"

Summary of Bias Assessment

Randomized Studies (n=5)

Low risk of bias: 3 studies (60%)

Some concerns: 2 studies (40%)

High risk of bias: 0 studies (0%)

Non-Randomized Studies (n=9)

Low risk of bias: 0 studies (0%)

Moderate risk of bias: 3 studies (33%)

High risk of bias: 6 studies (67%)
