# Supplementary figures and images for: Effects of swinging exercise on immune biomarkers: a systematic review and meta-analysis with machine learning-based identification of responder profiles
Source: Front Physiol. 2026 Feb 24;16:1694645. doi: 10.3389/fphys.2025.1694645 (PMC12973063; doi:10.3389/fphys.2025.1694645)

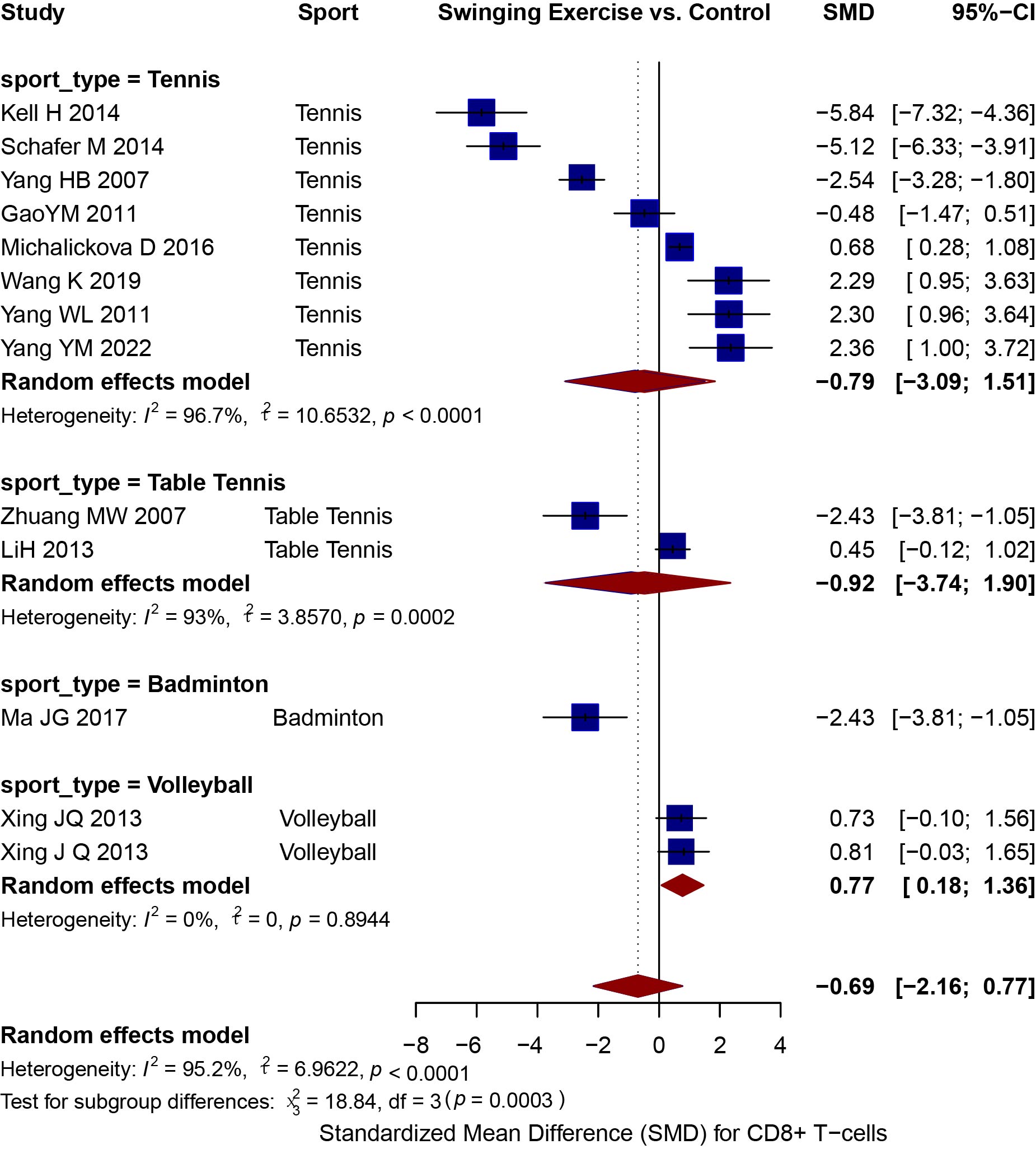

Supplement: Supplementary file 2 [file Presentation1.zip › Supplementary Figure S3.Effects on CD8+ T−cells.jpg]

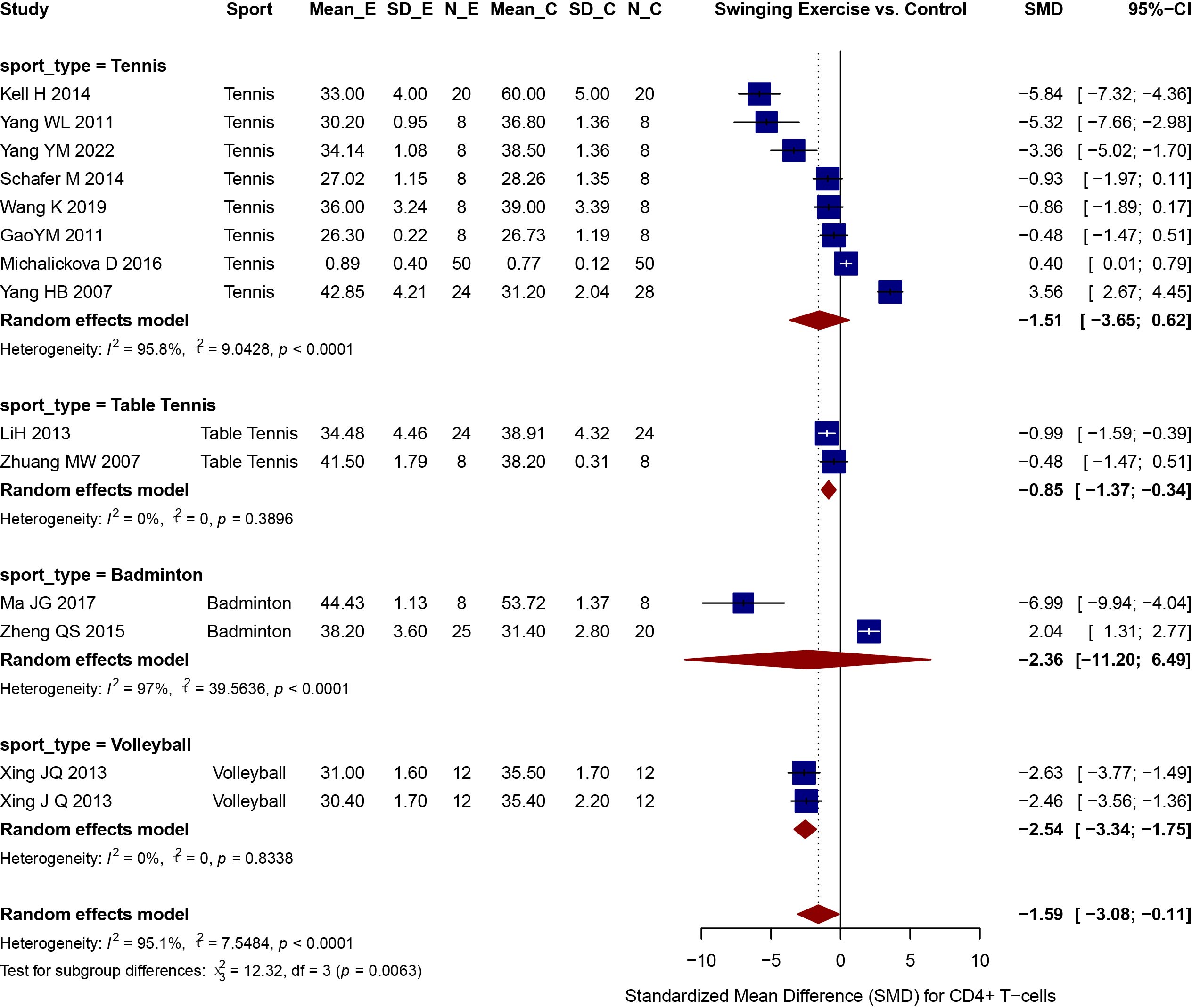

Supplement: Supplementary file 2 [file Presentation1.zip › supplementary figure2.CD4_Subgroup_Analysis.jpg]

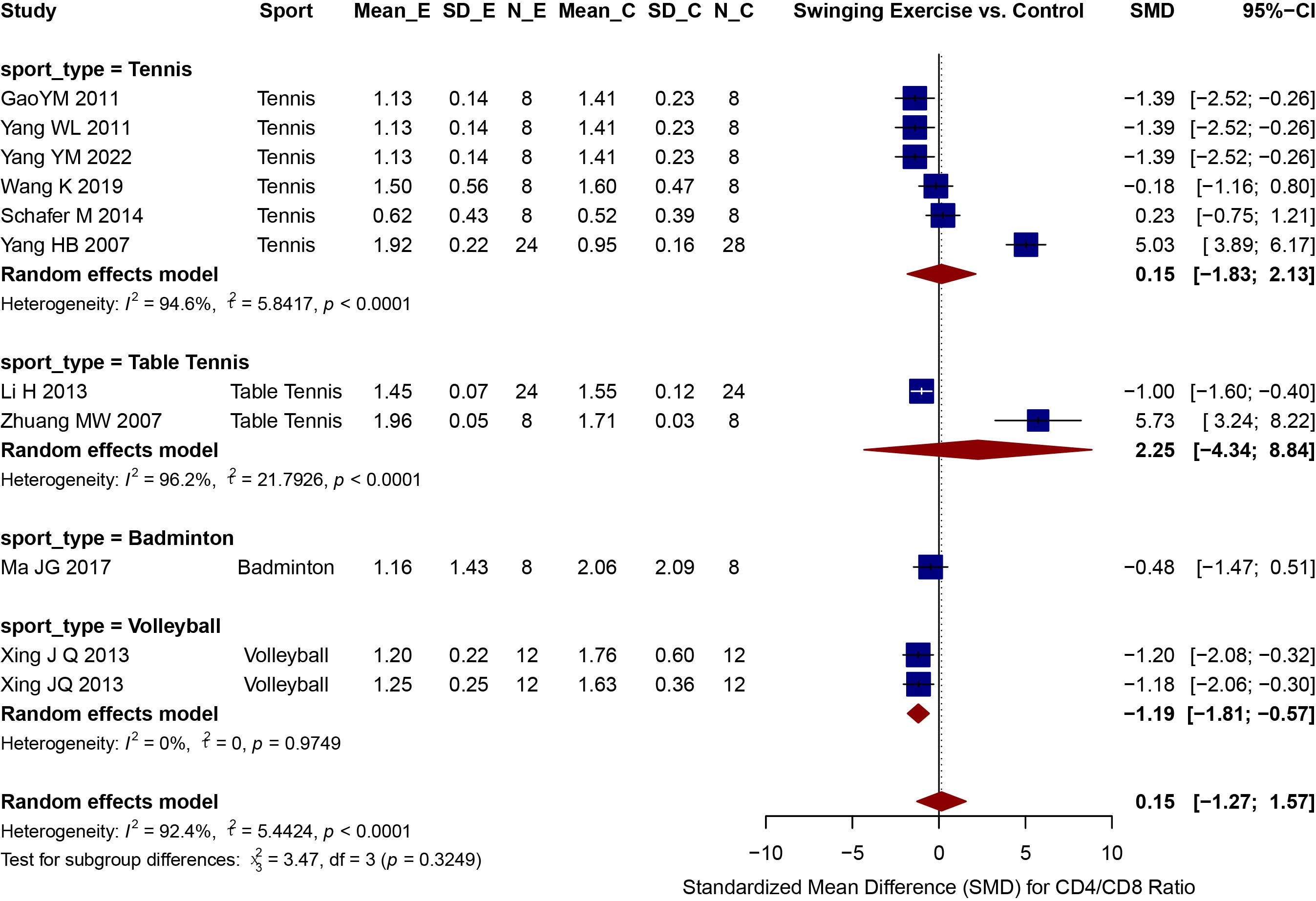

Supplement: Supplementary file 2 [file Presentation1.zip › Supplementary_Figure_S1_CD4_CD8_Subgroup_Analysis.jpg]
