# Supplementary material for: Effects of swinging exercise on immune biomarkers: a systematic review and meta-analysis with machine learning-based identification of responder profiles
Source: Front Physiol. 2026 Feb 24;16:1694645. doi: 10.3389/fphys.2025.1694645 (PMC12973063; doi:10.3389/fphys.2025.1694645)
Supplement: Supplementary file 3 [file Supplementaryfile3.doc]

# Supplementary file S3: GRADE Evidence Certainty Assessment

Detailed GRADE Assessment for Each Outcome

1. T-Cell Subsets (CD4+, CD8+, CD4+/CD8+ ratio)

Initial Grade: High (randomized trials)

Downgrading Factors:

Risk of Bias: -0 (Most studies showed low risk of bias)

Inconsistency: -1 (Substantial heterogeneity, I² = 68-75%)

Indirectness: -0 (Directly relevant population and interventions)

Imprecision: -0 (Confidence intervals exclude null effect)

Publication Bias: -0 (Funnel plot symmetrical, Egger's test P = 0.254)

Final Grade: ⊕⊕⊕◯ MODERATE

2. B-Cell Immunoglobulins (IgA, IgG)

Initial Grade: High (randomized trials)

Downgrading Factors:

Risk of Bias: -0 (Adequate randomization and allocation)

Inconsistency: -1 (Moderate heterogeneity, I² = 55-60%)

Indirectness: -0 (Directly relevant outcomes)

Imprecision: -1 (Wide confidence intervals, small sample size)

Publication Bias: -0 (Insufficient studies to assess)

Final Grade: ⊕⊕◯◯ LOW

3. Inflammatory Markers (TNF-α, IL-6)

Initial Grade: High (randomized trials)

Downgrading Factors:

Risk of Bias: -0 (Adequate methods)

Inconsistency: -2 (Very substantial heterogeneity, I² > 75%)

Indirectness: -0 (Directly relevant outcomes)

Imprecision: -1 (Very wide confidence intervals crossing null)

Publication Bias: -0 (Insufficient studies to assess)

Final Grade: ⊕⊕◯◯ LOW

4. Cardiorespiratory Markers (CK, LDH, BUN)

Initial Grade: High (randomized trials)

Downgrading Factors:

Risk of Bias: -0 (Adequate methods)

Inconsistency: -1 (Substantial heterogeneity, I² = 65-70%)

Indirectness: -0 (Directly relevant outcomes)

Imprecision: -2 (Very few studies, small sample sizes)

Publication Bias: -0 (Insufficient studies to assess)

Final Grade: ⊕◯◯◯ VERY LOW

GRADE Criteria Definitions

Factors for Downgrading Evidence:

Risk of Bias: Limitations in study design and execution

Inconsistency: Unexplained heterogeneity in results

Indirectness: Evidence not directly applicable to question

Imprecision: Wide confidence intervals or small sample sizes

Publication Bias: Evidence missing due to publication practices

Factors for Upgrading Evidence:

Large Effect Size: Large or very large magnitude of effect

Dose-response Gradient: Evidence of dose-response relationship

Plausible Confounding: All plausible confounding would reduce effect

GRADE Certainty Levels:

High (⊕⊕⊕⊕): Very confident that true effect is close to estimated effect

Moderate (⊕⊕⊕◯): Moderately confident in effect estimate

Low (⊕⊕◯◯): Limited confidence in effect estimate

Very Low (⊕◯◯◯): Very little confidence in effect estimate
