# Supplementary material for: Effects of swinging exercise on immune biomarkers: a systematic review and meta-analysis with machine learning-based identification of responder profiles
Source: Front Physiol. 2026 Feb 24;16:1694645. doi: 10.3389/fphys.2025.1694645 (PMC12973063; doi:10.3389/fphys.2025.1694645)
Supplement: Supplementary file 4 [file Supplementaryfile1.doc]

**Supplementary File S1: Complete Search Strategy**

1. PubMed Search Strategy

Search Date: January, 2025

Search Terms:

("table tennis"[MeSH] OR "tennis"[MeSH] OR "badminton"[MeSH] OR "volleyball"[MeSH] OR "racquet sports"[MeSH] OR "swinging sports"[tiab] OR "racket sports"[tiab])

AND

("T-lymphocytes"[MeSH] OR "T cells"[tiab] OR "CD4"[tiab] OR "CD8"[tiab] OR "CD3"[tiab] OR "T-cell subsets"[tiab])

AND

("B-lymphocytes"[MeSH] OR "B cells"[tiab] OR "immunoglobulins"[MeSH] OR "IgA"[tiab] OR "IgG"[tiab] OR "IgM"[tiab])

AND

("exercise"[MeSH] OR "exercise training"[tiab] OR "physical activity"[tiab] OR "athletic training"[tiab])

2. Web of Science Search Strategy

Search Date: January, 2025

Search Terms:

TS=(("table tennis" OR tennis OR badminton OR volleyball OR "racquet sports" OR "swinging sports" OR "racket sports")

AND

("T cell*" OR "T-lymphocyte*" OR CD4 OR CD8 OR CD3 OR "T-cell subset*")

AND

("B cell*" OR "B-lymphocyte*" OR immunoglobulin* OR IgA OR IgG OR IgM)

AND

(exercise OR "exercise training" OR "physical activity" OR "athletic training"))

3. Cochrane Library Search Strategy

Search Date: January, 2025

Search Terms:

("table tennis" OR tennis OR badminton OR volleyball OR "racquet sports" OR "swinging sports" OR "racket sports")

AND

("T cell" OR "T-lymphocyte" OR CD4 OR CD8 OR CD3 OR "T-cell subset")

AND

("B cell" OR "B-lymphocyte" OR immunoglobulin OR IgA OR IgG OR IgM)

AND

(exercise OR "exercise training" OR "physical activity" OR "athletic training")

4. Google Scholar Search Strategy

Search Date: January, 2025

Search Terms:

"table tennis" OR tennis OR badminton OR volleyball AND

T cell OR CD4 OR CD8 AND

B cell OR immunoglobulin OR IgA OR IgG OR IgM AND

exercise OR training

5. CNKI (China National Knowledge Infrastructure) Search Strategy

Search Date:January 2025

Chinese search formula:

SU=('Table Tennis' OR' Tennis' OR 'Badminton' OR 'Volleyball' OR 'Swing Sports')

AND

SU=('CT cell 'OR'CD4' OR'CD8 'OR'CD3' OR'CT lymphocyte ')

AND

SU=('Bell 'OR' Immunoglobulin 'OR' IgA 'OR' IgG 'OR' IgM ')

AND

SU=('Sports' OR 'Training' OR 'Physical Exercise')

Search Filters Applied:

Time Filter: No date restrictions

Language Filter: English and Chinese only

Publication Type: Original research articles only

Study Design: Clinical trials, randomized controlled trials, intervention studies

Boolean Operators Used:

AND: To combine different concepts

OR: To include synonymous terms within the same concept

NOT: Not used to ensure comprehensive retrieval

MeSH Terms Utilized:

Table Tennis [MeSH]

Tennis [MeSH]

Badminton [MeSH]

Volleyball [MeSH]

Racquet Sports [MeSH]

T-Lymphocytes [MeSH]

B-Lymphocytes [MeSH]

Immunoglobulins [MeSH]

Exercise [MeSH]

Search Validation:

Forward and backward citation searching performed for included studies

Reference lists of relevant reviews manually screened

Search strategy peer-reviewed by two independent information specialists
