# Supplementary material for: Effects of swinging exercise on immune biomarkers: a systematic review and meta-analysis with machine learning-based identification of responder profiles
Source: Front Physiol. 2026 Feb 24;16:1694645. doi: 10.3389/fphys.2025.1694645 (PMC12973063; doi:10.3389/fphys.2025.1694645)
Supplement: Supplementary file 2 [file Presentation1.zip › supplement fig legand.doc]

**Supplementary Figure S1.** Effects on CD4+/CD8+ T−cell Ratio

**Supplementary Figure S2.** Effects on CD4+ T−cells

**Supplementary Figure S3.** Effects on CD8+ T−cells
